# Predictive Modeling of Soft Stretchable Nanocomposites Using Recurrent Neural Networks

**DOI:** 10.3390/polym14235290

**Published:** 2022-12-03

**Authors:** Josué García-Ávila, Diego de Jesus Torres Serrato, Ciro A. Rodriguez, Adriana Vargas Martínez, Erick Ramírez Cedillo, J. Israel Martínez-López

**Affiliations:** 1 Tecnologico de Monterrey, Escuela de Ingeniería y Ciencias, Monterrey 64849, Mexico; 2 Department of Mechanical Engineering, Stanford University, Stanford, CA 94305-2004, USA; 3 DTU Nanolab, National Centre for Nano Fabrication and Characterization, Technical University of Denmark, 2800 Kongens Lyngby, Denmark; 4 Laboratorio Nacional de Manufactura Aditiva y Digital MADiT, Apodaca 66629, Mexico; 5 3D Factory, Ramon Treviño 1109, Monterrey 64580, Mexico; 6 Centro de Investigación Numericalc, 5 de mayo 912 Oriente, Monterrey 64000, Mexico

**Keywords:** data-driven, physics-based models, machine learning, recurrent neural networks, flexible electronics, nanocomposite, PDMS, SWCNTs

## Abstract

Human skin is characterized by rough, elastic, and uneven features that are difficult to recreate using conventional manufacturing technologies and rigid materials. The use of soft materials is a promising alternative to produce devices that mimic the tactile capabilities of biological tissues. Although previous studies have revealed the potential of fillers to modify the properties of composite materials, there is still a gap in modeling the conductivity and mechanical properties of these types of materials. While traditional Finite Element approximations can be used, these methodologies tend to be highly demanding of time and processing power. Instead of this approach, a data-driven learning-based approximation strategy can be used to generate prediction models via neural networks. This paper explores the fabrication of flexible nanocomposites using polydimethylsiloxane (PDMS) with different single-walled carbon nanotubes (SWCNTs) loadings (0.5, 1, and 1.5 wt.%). Simple Recurrent Neural Networks (SRNN), Long Short-Term Memory (LSTM), and Gated Recurrent Units (GRU) models were formulated, trained, and tested to obtain the predictive sequence data of out-of-plane quasistatic mechanical tests. Finally, the model learned is applied to a dynamic system using the Kelvin-Voight model and the phenomenon known as the bouncing ball. The best predictive results were achieved using a nonlinear activation function in the SRNN model implementing two units and 4000 epochs. These results suggest the feasibility of a hybrid approach of analogy-based learning and data-driven learning for the design and computational analysis of soft and stretchable nanocomposite materials.

## 1. Introduction

Flexible pressure sensors convert mechanical stimuli to electrical signals such as resistance, capacitance, or electrical potential. For resistive force sensing, a conductive material (polymers or metals) responds to an external force in arbitrary directions under compression or stretching deformation. A capacitive flexible pressure sensor is typically constructed as a parallel-plate capacitor with a dielectric polymer sandwiched between two flexible electrode layers [1]. Usually, resistive sensors have good sensitivity and are very sensitive to temperature, but they suffer from lower repeatability and high-power consumption [2,3]. Moreover, capacitive-based sensors have excellent sensitivity and high spatial resolution but are susceptible to electromagnetic interference, crosstalk, and parasitic capacitance [4]. Sensitivity is typically characterized by using the Gauge Factor (GF) or the fractional change in electrical resistance to the fractional change in length. Typically, the GF value of piezoresistive-enabled sensors is one or two orders of magnitude higher than in non-piezoresistive materials [5].

Technological advances over the past twenty years have combined formative, subtractive and additive manufacturing to produce complex composite devices within the micrometric scale [6,7]. The finesse of parts manufactured by these technologies can be applied to materials technology to produce devices with tunable properties. For example, carbon nanotubes can create thermal paths between adjacent carbon fibers and increase the thermal and electrical conductivity of composites [8,9]. The selection of resins that build up the composite matrix material depends on the compatibility with carbon and the adequate selection of processing conditions such as curing time, temperature, and pressure [10]. Typically, those parameters must be studied experimentally in the lab to develop procedures for a particular application. Further modeling can be performed experimentally (which can be expensive) or by employing numerical simulations. A recent review by Nurazzi et al. [11] covers a comprehensive CNT/polymer composite research summary.

While these numerical studies can save reagents and other materials, they still must tackle the high computation cost required for solving nonlinear and asymmetric models. Models based on physical principles are typically described by partial/ordinary differential equations (PDEs/ODE), where the description can be rigid and rely excessively on explicit assumptions. Combining neural networks with FEM can provide scenarios where the strengths of FEM (geometric flexibility and rich set of FE functions) and the flexibility of neural networks to express unknown functions are combined [12].

The study of the correlation between the processing parameters and the mechanical properties of materials are generally described experimentally. Neural network modeling is suitable for simulating correlations that are hard to describe by physical models. An important aspect of artificial neural networks is that a large amount of data is required for better prediction. The Artificial Neural Network or ANN models are among the most frequently employed machine learning models due to their performance, the proficiency to approximate complex nonlinear relations, and the availability of open-source code libraries [13]. Neural networks are control systems with determined connections between input and output parameters and allowable error deviations between the predictive value and the actual value calculated by the loss function [14]. Neural networks have been widely used for the identification of nonlinear dynamical systems and their state space models of different natures, such as biological neurons [15], oscillators [16], autonomous vehicles [17], organic molecules [18], and economic systems [19]. There is a potential for applying neural network architectures to obtain better predictive data modeling for a state-space model of soft materials. Furthermore, ANN can replace parts of constitutive material laws or use them as a surrogate for nonlinear materials [20].

The nonlinear constitutive modeling of composite materials remains a critical challenge for scenarios with nonlinear deformation or arbitrary loading conditions, considering the inability of standard forward neural networks to handle sequential information [21,22]. With the increasing complexity of composite microstructures, the nonlinear homogenized constitutive behavior at the macroscale is likely to be driven by one or multiple nonlinear mechanisms at the subscale [13], for instance, multi-walled carbon nanotubes can exhibit nonlinear electrical behavior [23].

Previously, the authors have investigated the nonlinear elastic response of RTV silicone and filler material SWCNTs (single-walled nanotubes) by fitting load-unloading curves using traditional parameter fitting algorithms for well-known models such as the Ogden-Roxburgh [24]. Viscoelasticity is a time-dependent mechanical behavior that can be easily observed/measured in soft materials and is dependent on the current state of deformation and deformation history. Theoretical models have been used to describe the behavior of viscoelastic materials using a spring and a damper to model the elastic and viscoelastic behaviors. Among these models, we can mention the Maxwell, Kelvin-Voight, Prony series, or Standard Linear Solid model [25]. Recurrent Neural Networks (RNN) are well suited to process this type of time series data and are designed to rely on historical information of sequential data. A hybrid approach of analogy-based learning and data-driven learning can provide means to adapt mechanistic models of soft materials with complex time-varying behaviors just as a dynamic system does.

Finite element results are highly accurate when the physical system is discretized correctly and can be used to train the machine learning model along with the input parameters. One way to achieve these conditions effectively is to train the models using large data sets produced by experimental data with well-defined standards. The learning process required can also be significantly expedited if the equations used to solve problems in several fields are the same (i.e., structural mechanics). Although some efforts have already been made in this direction [21,22,26], most studies on matching FEM and RNN are fragmentary. To the author’s knowledge, there is not a publicly available machine learning tool that can currently replace or outperform finite element simulators. However, research on this topic is relevant because the advance in artificial intelligence, machine learning, and neural networks can lead to the development of tailored materials from the starting point of desired functionality [27].

### 1.1. Analogy-Based Learning and Data-Driven Learning of Dynamic Mechanical Systems

A conventional dynamical system involves a state-space model that can be defined as a behavior in function of continuous-time t. The state of the system can be one, or a set of, different physical quantities (temperature, position, velocity, etc.) used to describe its behavior in any instant of time. This promising idea has been well received for the design of multi-joint systems such as ankle-foot prostheses. Additionally, there is an interest in using dynamics models to analyze the performance of multifunctional materials, such as flexible conductive materials, during large deformations. For simple problems, it is easy to find an analytical solution to describe the state of the system via governing equations and then to fit known parameters in advance. For many nonlinear dynamic mechanical systems, an analytical solution does not exist or is very difficult to obtain. As a result, mechanical analogies are used in the classic description to derive viscoelastic constitutive models.

**Figure 1:**
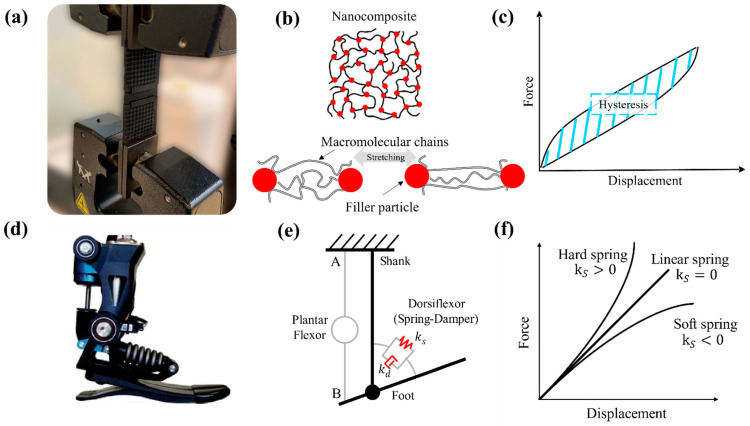
Flexible material (**a**) under stretching condition; (**b**) Cross-linked nanocomposite chains using spring-based interaction: (**c**) Uniaxial cycle response to show Mullins hysteresis effect behavior; (**d**) Powered ankle-foot prosthesis (image adapted from [28]); (**e**) Compliant dorsiflexor model with a spring-damper system in parallel configuration ($k_{s}$: elastic spring constant, $k_{d}$: dashpot viscosity constant; (**f**) Curves of restoring spring force.

For example, an analogy can be observed between a powered ankle–foot prosthesis using a variable nonlinear spring and the Mullins effect in filled soft materials. A nanocomposite under uniaxial deformation, as shown in Figure 1a, usually undergoes a stretching of the macromolecular chains attached to the filler particles as shown in Figure 1b. This deformation mechanism in soft materials presents dissipative energy or hysteresis in the force vs. displacement curves enveloped by the loading curve and the unloading curve, as shown in Figure 1c. Analogically, the inherent viscoelasticity of these types of materials can be assessed using the same fundamental mechanical descriptors of the dorsiflexion of a prosthesis as shown in Figure 1d using viscoelastic units (Hookean spring $\;k_{s}$ and the Newtonian damper $k_{d}$, see Figure 1e) that can be combined in series or parallel, and include nonlinearities in the mechanical behavior as shown in Figure 1f.

Data-driven learning-based approximation strategy can generate superior results with learned prediction models via neural networks and experimental data from a dynamic system. A recurrent neural network—or RNN—has several advantages, including the ability to process inputs of any length, the condition that the size of the model does not increase with the input, and that these models learn faster if the gradient tends to have a more drastic variation [29]. Researchers have shown promising results using RNN to predict the dynamic hysteresis of soft magnetic material [30], and nanocomposite piezo-resistive sensors fabricated from silicone rubber (Ecoflex) blended with carbon nanotubes (CNTs) [31]. On the other hand, Nagurka and Huang [32] and many others [33,34] subsequently analyzed the dynamics of a bouncing ball using a mass-spring-damper system analogy. The simple example of the bouncing ball clearly shows the presence of different deformation mechanisms acting on dynamically complex soft and stretchable objects. Other authors have used the damping-spring-mass, bouncing ball, and deep learning to provide a successful reduced-order model to describe the dissipative behavior of nonlinear phenomena [35]. Therefore, at the end of the manuscript, we present the bouncing ball experiment as numerical proof to model the dynamic system from experimental data trained by the RNN architectures.

### 1.2. Objective

In this paper, data-driven computation simulations using three classic recurrent neural networks (RNN) architectures and a one-step approximation method are employed for learning the input-output behavior of the dynamical viscoelastic response of soft nanocomposite materials. Physics-informed schemes are incorporated in the loss function to optimize the training and learning processes for the time-varying dynamics of nonlinear stress–strain and Mullins effect curves. The nanocomposite was synthesized to obtain flexible polydimethylsiloxane (PDMS) samples with single-walled carbon nanotubes (SWCNTs) as material filler. There is previous work within the research group of this article to manufacture molds using stereolithography to obtain well-defined geometric patterns of RTV-CNT composites [24]. For this work, we explore the concept further for PDMS and employ neural networks for the modeling.

**Figure 2:**
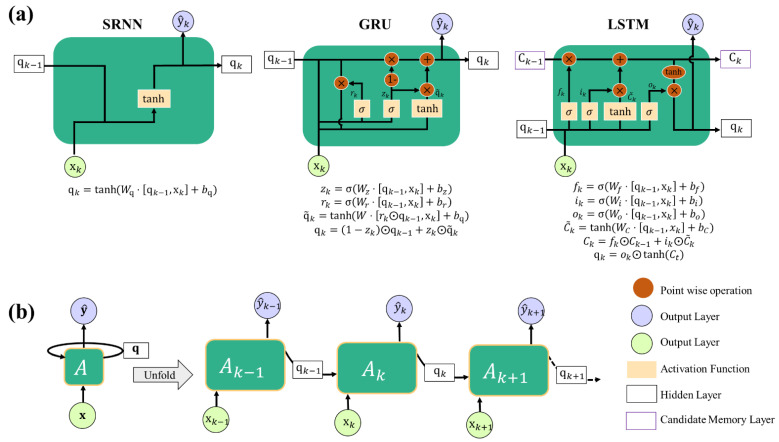
Three types of vanilla Recurrent Neural Network cells: (**a**) Simple Recurrent Neural Network (SRNN), Gate Recurrent Unit (GRU), and Long Short-Term Memory (LSTM); (**b**) an unfolded standard Recurrent Neural Network in repeating module: The right-hand side schematic is the unfolding version of neural network A through time. Here, x and $\hat{y}$ represent input, and the output vectors for the *k*-th state, while **q** represents the hidden state. This paper uses bold forms to represent vectors.

Three behavior conditions are considered to obtain the data sets: the hysteresis loops of the Mullins Effect, the uniaxial stress-strain curves, and two non-conventional tests based on the spring-damper system analogy. We performed a benchmark of the well-known vanilla recurrent neural network (RNN), see Figure 2a. These recurrent RNN structures have feedback loops in the recurrent layer and can transfer time dependence or maintain information in ‘memory’ over time through hidden units. In the case of SRNN, the hidden state at time step k is calculated based on the previous hidden state $q_{k-1}$ (containing information from the past) and the input at the current step $x_{k}$ as follows $q_{k}=\sigma \left(W\cdot \left[q_{k-1},x_{k}\right]+b\right)$. The function σ usually is a nonlinearity such as tanh, *W* are the weight matrices and *b* is the bias term. LSTMs and GRUs are a variant of RNN that solve the long-term memory or vanishing gradient problem of the SRNN by introducing new gates to control when information enters the memory, when it’s output, and when it’s forgotten defined here as *z* and *r* for GRUs and *i*, *o*, and *f* for LSTM. For simplicity, we illustrate in Figure 2b an RNN with only one hidden layer, i.e., one-stacked RNN, the equations of SRNN, LSTM, and GRU cells are provided below in Figure 2a. 

By using a neural network to approximate increments on the system and its surroundings we can avoid the governing equations to determine the behavior of soft material with nonlinear deformation. To assess the effectiveness of the proposed strategy, we performed out-of-plane non-conventional deflection tests for the maximum displacement and maximum Von mises stress using COMSOL Multiphysics without using hyperelastic model equations or fitting parameters.

## 2. Materials and Methods

### 2.1. Composite Films Sample Preparation

We followed a similar methodology developed and documented by the authors in a reported work [29]. For this paper we prepared nanocomposite samples using PDMS Sylgard 184 (Dow Corning, Midland, MI, USA) with ratio 10:1 and Tuball™ Matrix 601 SWCNTs nanotubes (OCSial, Columbus, OH, USA) at concentrations of 0.5, 1.0, and 1.5 wt.%. The nanocomposite was cast into 3D printed molds that do not inhibit curing to obtain 4 types of specimens as listed in Table 1.

**Figure 3:**
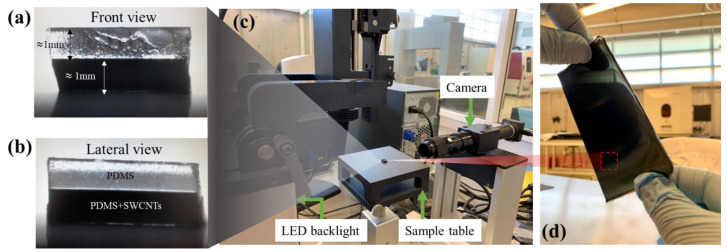
Doubled layer films (**a**) Front view; (**b**) Lateral view; (**c**) Setup for cross-sectional thickness inspection of film samples; (**d**) Translucent flexible film with minimum filler loading of 0.5 wt.%.

The double-layer films were poured into 91 mm × 91 mm commercial polycarbonate square containers, the approximate thickness of 1 mm (each layer, see Figure 3a) was controlled by measuring the volume poured into the containers, the bottom layer is 1.0 wt.% nanocomposite material and the top layer was PDMS as shown in Figure 3b, the pouring time between each layer had an intermediate time of 24 h, visual inspection was performed using an OCA 15EC equipment (DataPhysics Instruments GmbH, Filderstadt, Germany) to inspect the contours and layer thicknesses using backlight and the level surface of the equipment (see Figure 3c). The low filler concentrations and the homogeneity of the dispersion in the PDMS matrix were appreciable, showing that at such concentrations, the flexible membrane is still translucent across its surface, as shown in Figure 3d.

### 2.2. Testing Validation Method for Stretchable Materials

**Figure 4:**
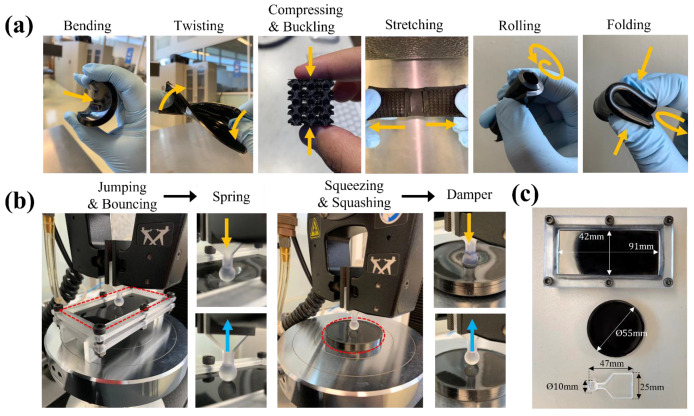
(**a**) Traditional in-plane deformation mechanisms for continuous or architected flexible materials; (**b**) deformation mechanisms inspired by out-of-plane indentation with unconventional boundary conditions that reveal elastic performance similar to spring or damper behavior descriptors; (**c**) custom-made fixture and indentation tip for out-of-plane deformation testing.

Flexible nonlinear and hyperelastic materials cannot be tested thoroughly with uniaxial tension testing because they exhibit different behavior under different deformation states. It is feasible to state that flexible materials (continuous or architected) present at least nine deformation mechanisms (see Figure 4). Although the typical deformation mechanisms are mostly well understood (Figure 4a), in this paper, we focus on deformation mechanisms primarily determined by the boundary conditions on flexible and stretchable materials (see Figure 4b). Squashing behavior as a descriptor of deformation in soft textured materials under indentation conditions has been reported previously [36]. The change in the shape of a generic bulk material may be due to other deformation mechanisms such as ripping, shearing, tearing, sticking, pushing, poking, sliding, pushing, clenching, grinding, or pulling. Therefore, it is helpful to perform mechanical performance tests beyond the standard uniaxial tension or compression mechanical tests to fully capture the dynamic or static response of a soft material. State-of-the-art on unconventional mechanical testing shows that there are multi-axial testing platforms (i.e., biaxial testing) and out-of-plane testing based on indentation (i.e., small punch testing) [37]. Our set-up experiment is based on a the punching device that characterizes the behavior of a material under biaxial tension based on testing standard ASTM F2183 [38]. This assessment uses a spherical punch to press a disk specimen held by an outer edge. The results are useful to characterize the biaxial out-plane stress-strain response of the material, and for validation of the hyperelastic model developed solely from the in-plane uniaxial approach. The spherical indenter (10 mm diameter), the custom-made fixture, and the tensile strength (dogbone) molds were 3D printed using a benchtop Form 3 additive manufacturing equipment (Formlabs, Somerville, OH, USA) (see Figure 4c).

The uniaxial tensile tests were carried out using Type 1A dog bone shape samples with an overall length of 100 mm and a 3 mm thickness following the standard ASTM D412-16 (2021) [39]. A universal machine (3365, INSTRON, Norwood, MA, USA) equipped with a 50 kN load cell was used. Three specimens of each type of continuous pure PDMS material and assessed composition (Sample I, Sample II, and Sample III) were loaded axially and monotonically at a speed deformation of 0.3 mm/s until complete failure. Next, loading-unloading uniaxial cyclic tests were performed with a maximum strain level ε=0.6  considering a 300 mm/min rate for 10 first continuous cycles to observe the stability of mechanical softening.

### 2.3. Coupling RNN with Mechanical Models

The different viscoelastic phenomena that constitute the behavior of flexible materials are classically studied separately. To model global behavior, it is necessary to combine approaches. Based on numerical analogies with the behavior of dynamical systems, recurrent neural network (RNN) architectures approximate the nonlinear mechanical behavior of soft nanocomposite materials. However, modeling techniques based on neural networks must consider the choice of efficient and compatible sub-models with few parameters for each phenomenon. The present work uses the following two essential mechanical sub-models:

1. The first sub-model is the generalized Kelvin-Voigt equations viscoelastic model, which can have a nonlinear spring in parallel with a nonlinearly viscous dashpot through $\varepsilon =f\left(\sigma_{s}\right),\;\;\dot{\varepsilon \;}=g\left(\sigma_{d}\right),\;\;\sigma =\sigma_{d}+\sigma_{s}$, where f and g could be nonlinear functions, $\sigma_{s}$ and $\sigma_{d}$ are the stresses in the spring and dashpot, respectively, σ is the total stress. The fractional-order derivative that describes this analogous mass-spring-damper system is $m\ddot{d}+k_{d}\dot{d}+k_{s}d=F$, where d denotes the deformation that we can obtain from uniaxial tests.
2. The second sub-model focuses on the behavior of hysteresis under loading conditions to define f and g. That is, in a viscoelastic element such as a damper the dissipated energy is expected to be higher, while in an elastic element, such as a spring, the elastic energy is expected to be higher. As the elastic and dissipated energy depend on the loading process, then, two deformation mechanisms inspired by out-of-plane indentation were used with unconventional boundary conditions that reveal the elastic and dissipative behavior of the nanocomposite similar to the behavior of springs or dampers.

**Figure 5:**
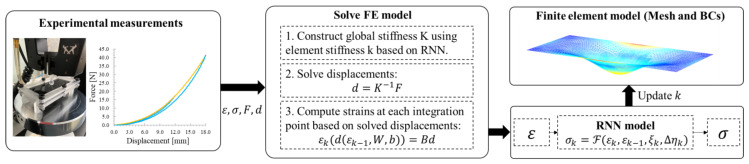
The framework of RNN–FE coupled approach based on experimental data (loading shown in yellow and unloading shown in blue).

The above sub-models are compatible with a numerical simulation known as a bouncing ball. The bouncing ball problem is a simple experiment that illustrates complex deterministic dynamical systems associated with energy losses using a damper-spring system, in this work this allows us to validate the analogy between the state–space model with the recurrent neural network. On the other hand, the RNN-coupled FE model’s general framework for learning a constitutive law is shown in Figure 5. The proposal RNN model has the form $\sigma_{k}=F\left(\varepsilon_{k},\varepsilon_{k-1},\xi_{k},\Delta \eta_{k}\right)$, where σ is stress, ε  is strain, and the subscript k and k−1 denote the current and previous load increments. $\zeta_{k}$ and $\Delta \eta_{k}$ are the internal variables defined as $\zeta n=\sigma_{k-1}\varepsilon_{k-1}$ and $\Delta \eta_{k}=\sigma_{k=1}\Delta \varepsilon_{k}$. The $\zeta_{k}$ implies its previous state along the equilibrium path by its energy quantity and $\Delta \eta_{k}$ implies the direction for the next load step along the equilibrium path. The RNN–FE model receives the measurable data (i.e., Force F, displacement d) from experiments. The global stiffness matrix K and the strain-displacement matrix B make up the standard 2D finite element method (FEM). Using the stiffness matrix created by the RNN model, the RNN–FE model first solves the displacements at each loading step.

### 2.4. Fundamental System Identification and RNN Analogy

First, the description of how the architecture of the recurrent neural network and the dynamic system might be compared follows. It is possible to convert nonlinear models to a linear model (in a small region around the equilibrium point), assuming a linear time-invariant system without loss of generality. We consider a continuous-time linear state–space model with n states, m inputs, and r outputs proposed as:

(1) $$\dot{x}\left(t\right)=Ax\left(t\right)+Bu\left(t\right)\;$$

(2) $$y\left(t\right)=Cx\left(t\right)+Du\left(t\right)$$

where at time t≥0, $x\in {\mathbb{R}}^{n}\;$ is the state vector (internal system memory), $u\in {\mathbb{R}}^{m}$ is the control input vector acting on the system, and $y\in {\mathbb{R}}^{r}$ is an observable (measured) output vector. The matrices $A\in {\mathbb{R}}^{n\times n}$ (system matrix), $B\in {\mathbb{R}}^{n\times m}$ (input matrix), $C\in {\mathbb{R}}^{r\times n}$ (output matrix), and $D\in {\mathbb{R}}^{r\times m}$ (feedthrough matrix) are real state–space matrices and have compatible dimensions, where m, n, r integers are positive numbers (n is also often called the order of the system). Similarly, the discrete-time version of the previous model has the following form:

(3) $$x_{k+1}=\tilde{A}x_{k}+\tilde{B}u_{k}\;$$

(4) $$y_{k}=\tilde{C}x_{k}+\tilde{D}u_{k}$$

where k is a discrete-time instant. That is, the approximations of the original state are made at the time t=kh, where h is a sampling period or discretization step. On the other hand, the RNN architectures such as Simple Recurrent Neural Networks (SRNN) have the following mathematical expression:

(5) $$q_{k}=\sigma_{q}\left(Lq_{k-1}+Ep_{k}+z\right)\;$$

(6) $$v_{k}=\sigma_{u}\left(Pq_{k}+g\right)$$

where $q_{k}$ is a hidden layer vector, $p_{k}$ is a Neural Network (NN) input vector, $v_{k}$ is an NN output vector, z and g are vectors of NN parameters, L, E, and P are matrices consisting of NN parameters, and $\sigma_{q}$, $\sigma_{v}$ are vectors activation function, and k is discrete time. The SRNN model described by Equations (5) and (6) resembles the state–space model described by Equations (3) and (4) when activation functions are linear, and the parameter vectors **z** and **g** are zero. Therefore, in some ways, an SRNN can be viewed as the traditional linear state–space model and vice versa.

Hence, our main objective is to train the parameters of RNN such that trained networks produce the input-output behavior of the discrete-time state–space model defined by Equation (2).

### 2.5. Baseline Numerical Mechanical Model and One-Step Approximation

Instead of training the discrete signal directly using RNN, the neural network in this paper parameterizes the derivative of structural states with respect to time. For a mass-spring-damper system, the equilibrium equation using Newton’s second law of motion in terms of the fractional time derivatives can be written as:

(7) $$m\ddot{d}+k_{d}\dot{d}+k_{s}d=F$$

where m is the mass, d is a displacement from the equilibrium point, $k_{d}$ and $k_{s}$ are viscous damping and spring coefficients and F is the external control force. Using the state–space variables $x^{\left(1\right)}=d$ and $x^{\left(2\right)}=\dot{d}$. Hence, ${\dot{x}}^{\left(1\right)}=\dot{d}=x^{\left(2\right)}$ and ${\ddot{x}}^{\left(1\right)}=\ddot{d}=-\frac{k_{d}}{m}\dot{d}-\frac{k_{s}}{m}d+\frac{1}{m}F=-\frac{k_{d}}{m}x^{\left(2\right)}-\frac{k_{s}}{m}x^{\left(1\right)}+\frac{1}{m}F$. So, the model defined by Equation (3) can be rewritten as the following state equation:

(8) $$\underset{\dot{x}}{\underbrace{\left[\begin{array}{c} {\dot{x}}^{\left(1\right)}\\ {\dot{x}}^{\left(2\right)} \end{array}\right]}}=\underset{A}{\underbrace{\left[\begin{array}{cc} 0 & 1\\ -\frac{k_{s}}{m} & -\frac{k_{d}}{m} \end{array}\right]}}\underset{x}{\underbrace{\left[\begin{array}{c} x^{\left(1\right)}\\ x^{\left(2\right)} \end{array}\right]}}+\underset{\dot{B}}{\underbrace{\left[\begin{array}{c} 0\\ \frac{1}{m} \end{array}\right]}}\underset{u}{\underbrace{F}}$$

The position vector d (state variable $x^{\left(1\right)}$) is the only one that can be directly measured. Hence, the output equation takes the matrix form:

(9) $$\underset{y}{\underbrace{d}}=\underset{C}{\underbrace{\left[\begin{array}{cc} 0 & 1 \end{array}\right]}}\underset{x}{\underbrace{\left[\begin{array}{c} x^{\left(1\right)}\\ x^{\left(2\right)} \end{array}\right]}}$$

The state–space model defined by Equations (5) and (6) is in the continuous-time domain. From the machine learning perspective, this is not convenient, so it is necessary to obtain the representation in the discrete-time domain. The backward Euler method was used to transform it into the discrete-time domain. Using this one-step approximation, we obtain:

(10) $$x_{k}=\tilde{A}x_{k-1}+\tilde{B}u_{k-1}$$

where $\tilde{A}={\left(I-hA\right)}^{-1}$ and $\tilde{B}=h\tilde{A}B$, both discrete matrix representation, and the output equation remains unchanged, and form as:

(11) $$y_{k}=\tilde{C}x_{k}$$

The discrete representations of the system defined in Equations (8) and (9) are sufficiently convenient to define the estimation model. Note the similarities between the state model defined above and the Simple Recurrent Neural Network described in the previous section. First, the recursive relationship of the hidden layer vector (q) and the state of the system (x), and the presence of input vectors (p and u) and output vectors (v, y), see Table 2. This highlights the importance of establishing a comparison between the neural network equations and the state–space system.

### 2.6. Data Sets Experimental Data and Network Setup

In order to train, fit, and learn the neural network parameters (L,E,z,g) based on experimental data, the physical sequence force observable input vector $u:\left\{u_{0},u_{1},\ldots ,u_{N}\right\}$ (and an initial state of the system) is required as input data, hence, it should produce the predictable sequence of output vector data $\hat{y}:\left\{{\hat{y}}_{0},{\hat{y}}_{1},\ldots ,{\hat{y}}_{N}\right\}$ that accurately approximates the output sequence $y:\left\{y_{0},y_{1},\ldots ,y_{N}\right\}$ of the real system. In other words, the main objective is to use a physics-driven model which takes an initial condition $x_{0}$ at time $t_{0}$ and produces an accurate prediction $\hat{x}$ of the actual state x such that $\hat{x}\left(t;x_{0}\right)\approx x\left(t;x_{0}\right)$ as much as possible. For the application of predicting state variables on a stress–strain curve that this manuscript aims to solve, the data set is defined as:

(12) $$S=\left\{x_{k}^{\left(i\right)},x_{k-1}^{\left(i\right)};u_{k}^{\left(i\right)},h_{k}\right\},\;i=0,\ldots ,N$$

where N is the length of sampled data pairs and $x_{k}^{\left(i\right)}$ denotes the i:th state variable **x** in the k:th data pair, and $x_{k-1}^{\left(i\right)}$ is the pertaining state variable one discrete time-step h. The force $u_{k}^{\left(i\right)}$ is the force that is acting on the system at the k:th time point. The goal is to make the L loss function adequately small which is achieved through training the NN parameters. Here, the Mean Squared Error (MSE) function L is our loss function (also known as the cost function) defined as:

(13) $$L\left(\hat{y},y\right)=\frac{1}{N}\overset{N}{\underset{i=1}{\sum }}{\left({\hat{y}}^{\left(i\right)}-y^{\left(i\right)}\right)}^{2}$$

In general, accuracy and loss are the two best-known metrics for neural network models, but accuracy is a valid metric of evaluation only for classification problems. The model addressed in this manuscript is a time-series-type regression problem, and therefore it is not possible to compute accuracy.

### 2.7. Coupled Discrete Numerical Simulation Framework

The full numerical simulation was developed in the computational machine with 16 GB RAM, 1 TB SSD, and Microsoft Windows 11 version 21H2 operating system (Redmond, WA, USA). The virtual environment Jupyter Notebook version 6.4.11 from NumFOCUS (Austin, TX, USA) was used. In the integrated framework, we coupled using MPh 1.2.0, a python scripting interface created by John Hennig as Open-Source software, to access the COMSOL API. We implemented a custom programming code using Python 3.10 from Python Software Foundation (Beaverton, OR, USA) installed in an Anaconda (version 1.7.2) environment from Anaconda Incorporated (Austin, TX, USA) to compute the discrete data sample from continuous-time system response via the backward Euler method with N=200 simulation time steps and sampling period (discretization step) h=0.01 s. The code was used to generate training, validation, and test data for three NN methods via Keras [40] (deep learning API) from TensorFlow open-source platform created by François Chollet. The network setup is a fully connected neural network with several unit cell conditions. Each unit cell architecture also has the variant sigmoid activation function $f\left(x\right)=\tan h\left(x\right)$. The time taken to train the artificial neural network depended on the quantity of data, the number of hidden layers, and the number of epochs. Experimental data are from uniaxial mechanical tests on 20 specimens (5 of each type listed in Table 1), 12 uniaxial loading and unloading specimens (3 of each type listed in Table 1), and 6 out-of-plane mechanical test specimens (3 tests in jumping/bouncing condition and 3 tests in squeezing/squashing condition).

## 3. Results and Discussion

### 3.1. Stress–Strain Behavior, Mullins Effect, and Strain Energy

The elastic energy ($U_{E}$), dissipated energy ($U_{D}$), and input total energy ($U_{T}$) of each loading–unloading cycle for the PDMS-SWCNTs samples were calculated as:

(14) $$U=\int_{x_{1}}^{x_{2}}Fdx$$

(15) $$U_{T}=U_{D}+U_{E},$$

**Figure 6:**
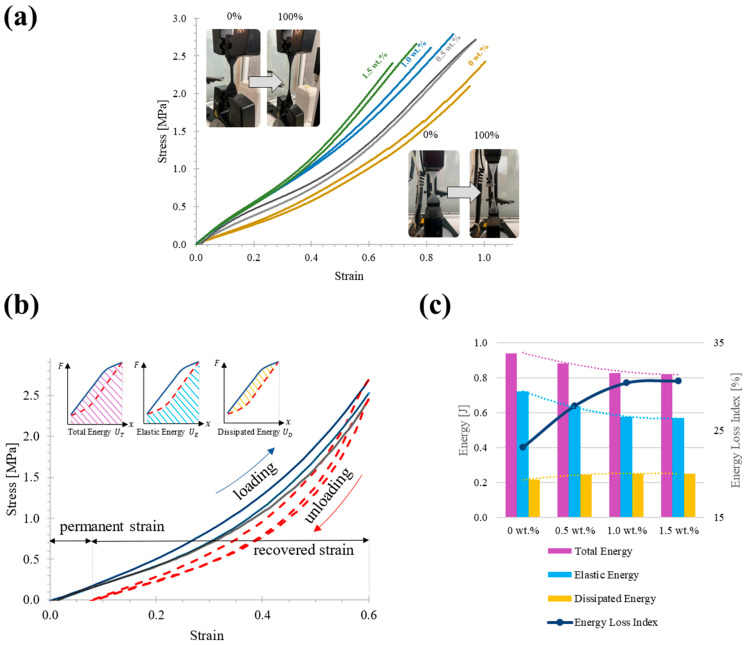
Experimental data for (**a**) Uniaxial static test until failure of the four types of specimens with different percentages of filler composition for 1.5 wt.% (green), 1.0 wt.% (blue), 0.5 wt.% (gray), 0 wt.% (olive); (**b**) Loading–unloading test showing the hysteresis behavior during nonlinear elastic deformation, as well as the permanent strain present in the nanocomposite material (continuous lines are during loading and dashed lines are during unloading); (**c**) Trend graph of the calculation of the three types of energy presented during the cyclic-to-cycle tests performed.

Figure 6a shows that the stress-strain and hysteresis curves of the three carbon nanotubes and PDMS combinations have nonlinear trends. The elastic energy ($U_{E}$) stored in the nanocomposite from elastic deformation is released during deformation recovery work. The dissipated energy ($U_{D}$) includes plastic strain energy which generates a permanent strain in the flexible material (see Figure 6b). The energy loss index refers to the ratio of the total energy accumulated to the strain energy dissipated in a uniaxial loading test. The experimental data were further processed as $\frac{U_{D}}{U_{T}}\times 100$ (see Figure 6c).

While quasistatic uniaxial tests allow determining of the influence of nanocomposite stiffness increase by varying the filler loads, as shown in Figure 6a, there is currently a debate on the influence of these fillers in cyclic loading and unloading tests where hysteresis behavior is present as a result of energy losses beyond the elastic behavior [41]. In practice, it is common to find strain-softening models that fit the mechanical hysteresis curves using parameterized equations. However, differences in fullness percentage are negligible (curves show remarkably similar characteristics, Figure 6b). In this section, we attempt a more quantitative analysis of the data, starting with a numerical calculation of the elastic energies encompassed by these curves that allow us to quantify the energy that can no longer be recovered by the well-known Mullins effect, according to Figure 6c the elastic energy tends to reduce as the percentage of filling increases under uniaxial loading condition, the marked trend of the loss energy index evidences the nonlinearity in the trend that is not possible to identify in the loading and unloading curves, these data are essential for the RNN model to learn the nonlinearity of the functions f  and g of the Kelvin-Voigt viscoelastic model.

**Figure 7:**
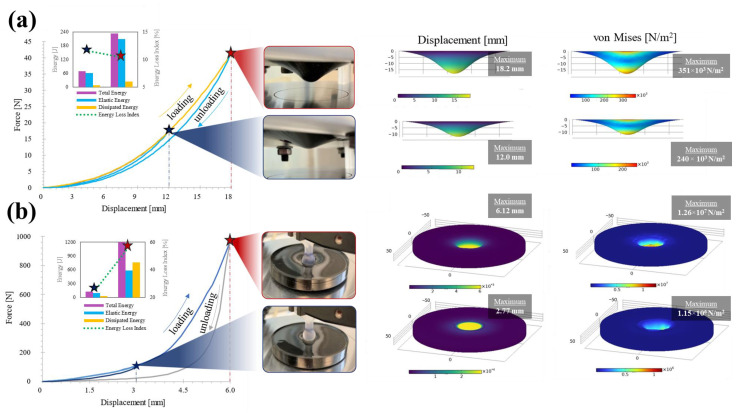
Non-conventional mechanical tests to demonstrate nonlinear behavior and energy losses in flexible materials under various nonlinear mechanical deformation conditions; (**a**) Spring-driven deformation, (**b**) Damper-driven deformation.

The out-of-plane mechanical test data is relevant complementary data for the neural network training model as it allows conditions to be obtained under unconventional mechanical performance (see Figure 7a). A mechanical test dominated by jumping and bouncing shows a highly deformable material with relatively low application forces and which easily recovers its original state with energy losses at around 10% of all potential strain energy, as seen in Figure 7a. In contrast, a squashing and squeezing dominated condition reflects high energy hysteresis behavior with an exponential trend as the strain in the material increases, as illustrated in Figure 7b. The matching FEM simulation was coupled numerically with input data from the elastic tests and complementary cyclic tests without using hyperelastic model equations or fitting parameters.

### 3.2. Nanocomposite Ball Dynamics Tuning Experimental Data

The dynamic mechanical properties of a vertically dropped hollow ball are studied to investigate the accuracy and efficiency of the proposed definitive data-driven method. The elastic shell of the ball is assumed to be four layers with a thickness of 0.5 mm for each one, one layer of PDMS, and one layer for each loading concentration of nanocomposite (0.5 wt.%, 1 wt.%, and 1.5 wt.%). Next, the main parameters of the bouncing ball are estimated from experimental data using a deep learning process to obtain an equivalent virtual simulation configuration. Previously, this model has been tested using discretization techniques and traditional computational optimizations with promising results [42]. The training process considers the energy loss and elastic energy from experimental data to obtain a closed-loop simulation. The governing equation of motion of the bouncing ball dynamics in the instant impact with the ground is described using Equation (4), where the *F* is the instant force gravity of the ball with initial conditions of $x_{0}=0$ and ${\dot{x}}_{0}=-v_{0}$, and $v_{0}$ is the impact velocity just before the impact. The main problem is that the bounce mechanical behavior, involving nonlinear deformation, restitution, energy loss, and then rebound, requires an underdamped solution. The other states of the system (before impact and the steady solution) are trivial solutions.

**Figure 8:**
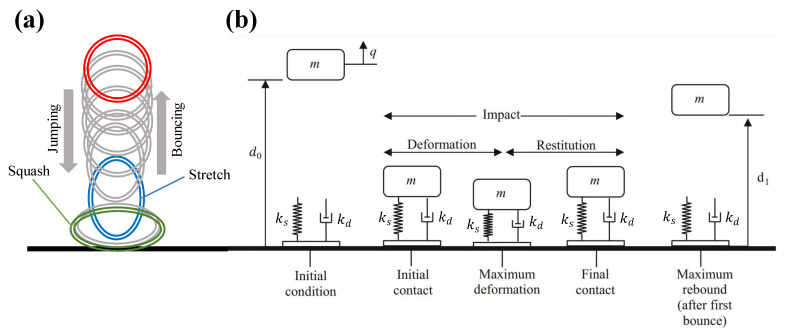
(**a**). Schematic representation of the deformation mechanism of bounding stretchable ball (a cross-section of the ball is shown); (**b**) A mass-spring-damper model of a bouncing ball showing phases of the first cycle (figure adapted and licensed from [42]).

The contact spring is used for the bouncing at impact and the contact damper for is for the squashing energy dissipation. It is assumed that there is no air friction or other energy loss that cannot be attributed to the spring-damper system. Different regimes should be considered before, during, and after the impact. The graphs in Figure 8a show the free fall and spring rebound. The vertical deformation and energy loss rates of the stretchable object were well explained by the spring-damper model. This model will be conceptually illustrated in Figure 8b using a nonlinear spring and a nonlinear viscous damper in parallel configuration (Kelvin-Voight viscoelastic model).

Data included in the Supplementary Material (Figure S1, see Supplementary Materials) show that the capability of the predicted output to match the real reference input between *p* = 8 and *p* = 64 is clear but comparing *p* = 32 and *p* = 64 using only the real reference output curves and the predicted output curve is not entirely obvious. Therefore, we provide the loss function curves, in which it is observed that using *p* = 64 in SRNN, GRU, and LSTM the function decays rapidly to very low values after roughly 10 epochs. In contrast, for *p* = 32 the epochs increase to a range of 20–40. However, increasing the number of units improves the prediction but only to a certain point, and further addition of units can actually harm the model’s performance. A clear example is the instabilities observed at the end of the SRNN loss function using *p* = 64 which results in a worse prediction curve fit than that achieved using *p* = 32.

**Figure 9:**
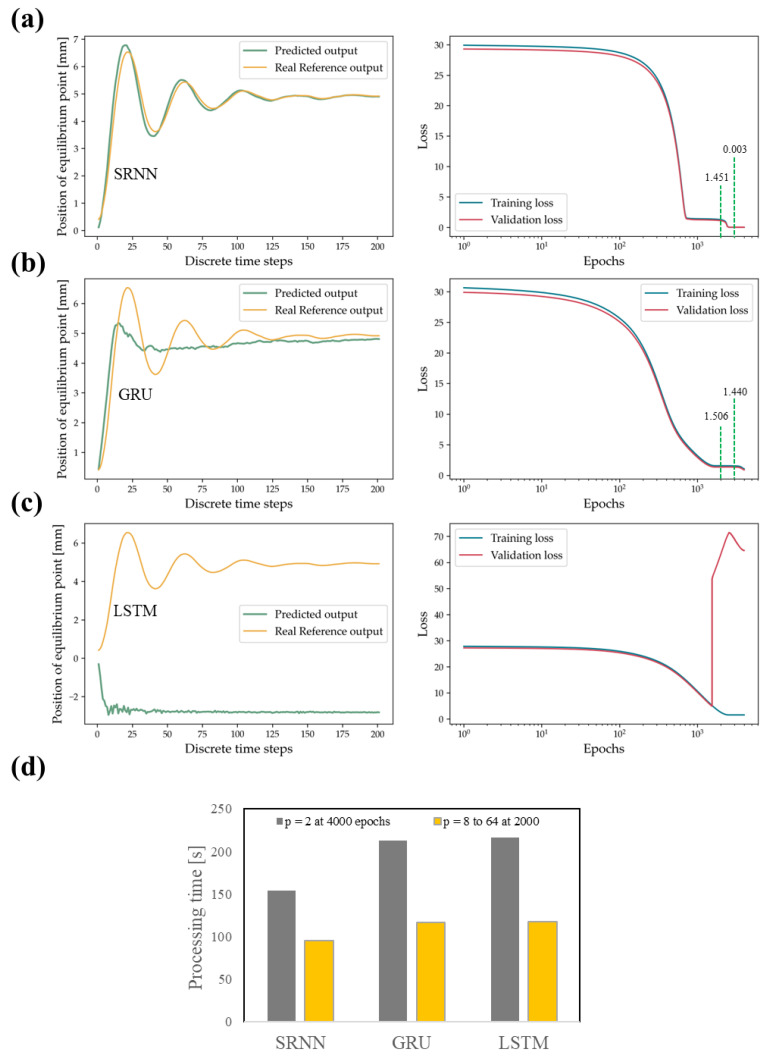
Prediction performance, training, and validation losses for modified neural networks; (**a**) SRNN, (**b**) GRU, (**c**) LSTM architectures and (**d**) Comparison of processing times using 2 units and 4000 epochs or 8–64 units and 2000 epochs.

These loss plots allow us to identify three common dynamics that are likely to be observed in learning curves: underfit, overfit, and good fit. A good fit is identified by a training and validation loss that decreases to the point of stability with a minimal gap between the two final loss values. A learning curve plot shows a good fit if the training loss plot decreases towards stability or the validation loss plot decreases to the point of stability and has a small gap with the training loss. It should be noted that the training was terminated after 2000 epochs, and the effect of overfitting was not found, in other words, if the gap between the performance in the training data and the test data is very wide, it means that, effectively, our model is overfitting, i.e., memorizing, not learning. A larger unit number permits a higher-order model for estimating the system and hence increasing the number of epochs also allows for better outcomes. The results of the Supplementary Materials incorporated the bias ‘b’ into the output function. For Figure 9, the bias was applied only in the activation functions. Each neuron is characterized by its weight, activation function, and bias. If there is any error during the prediction by the function, bias can be added to the output values to obtain the true values.

Typically, GRU and LSTM are used to avoid the vanishing gradient problem in cases where the sequential training data is redundant, and the memory loss is propagated over time. However, these NN use more activation functions than SRNN. Figure 9a shows that the activation function of the SRNN architecture (tanh) while reducing the neuron units to *p* = 2 gives a better fit prediction in comparison to GRU and LSTM models. Furthermore, the LSTM architecture presented as overfitting in the data due to the small number of neuron units *p* and its complex architecture as shown in Figure 9c. If we observe the GRU and SRNN loss graphs in Figure 9a,b, we will see that both configurations allow very low and similar loss values to be obtained, showing that this stability value (position of equilibrium point) is the first value that the system learns. On the Supplementary Materials, it is observed that for *p* = 8 the models predict a value close to the position of the equilibrium point.

For this specific case, the equilibrium position is extremely influential in the loss function and therefore GRU and SRNN are models with good fit. However, if one looks closely at the loss function in Figure 9a there is a slight decrease of the loss at the end which allows us to adjust the rest of the data of the oscillating curve around the equilibrium point. The values of loss of SRNN and GRU in 2000 and 3000 epochs shown in Figure 9a,b show that the fit of the oscillations around the equilibrium point has an associated value of roughly 1.4. Varying between 8 and 64 units has negligible impact on processing time, however processing the learning model at twice the number of epochs requires significantly more time, but the SRNN model requires significantly less processing time compared to the other two architectures due to the simplicity of its architecture, see Figure 9d. The Supplementary Materials is intended to test the performance of the system using half the number of epochs (2000) as the test presented in Figure 8 (4000). That is, Figure 9c shows LSTM with 4000 epochs and *p* = 2. However, LSTM requires at least *p* = 8 to obtain the stability value of the equilibrium point as shown in the Supplementary Materials.

## 4. Conclusions and Future Work

We presented several neural network (NN) structures to approximate the nonlinear mechanical behavior of soft nanocomposite materials based on an analogy with the behavior of dynamic systems, using experimental elastic deformation data from static tensile testing and loading and unloading. The NN structures are based on Recurrent Neural Networks (RNN), Gated Recurrent Unit (GRU), and Long Short-Term Memory (LSTM). Derivations of the simplest case were presented to show the analogy of state–space models and mechanical dynamical systems. Once successfully trained, the methods produce discrete dynamical systems that approximate the unknown underlying governing equations of the nonlinear deformation of nanocomposite material.

The fundamental challenge with the approach proposed in this work is that it requires knowledge of a beginning condition to be applied. In real-world applications, the beginning state of a dynamic system is rarely known. However, if the system is stable, the initial state can be ignored because the effect of the initial condition is neglected on the steady state. An alternative to explore in the future is to develop an autoregressive-exogenous (ARX) model. This model predicts the system’s output only based on previous inputs and outputs.

The solution of a general mechanical dynamical system can be estimated using only the trained neural network and a state–space variables. When training the neural network operator to approximate the effective output data, the analytical and experimental solutions of test mechanical configurations are used to produce the training data on which the network can be trained.

We successfully described the concept of neural networks and the surrounding concepts without the necessity for a known governing equation when defining the system to mechanically represent the behavior of a soft material with nonlinear deformation. A broad mechanical dynamical solution of the system can thus be approximated using only a trained neural network and state–space variables in complex deformation phenomena such as the bouncing ball. Efficiency in other deformation mechanisms can be explored to improve our compression of soft materials and their energy losses. Loading concentrations affect the elastic and dissipative energy during the loading and unloading cycles. However, the training data used covers a limited range of 0 to 1.5% wt. for the specific case of SWCNTs nanotubes, so validating their effectiveness with other types of compositions or constituents requires future research efforts.

## Figures and Tables

**Table 1:** Composite material preparation.

Component	Sample I Weight, g (1.5 wt.%)	Sample II Weight, g (1 wt.%)	Sample III Weight, g (0.5 wt.%)	Sample IV Weight, g (0 wt.%)
SWCNTs Tuball™ Matrix 601	1.8	1.2	0.6	0
Sylgard 184 part A	107.45	108	108.54	109.09
Sylgard 184 part B	10.745	10.8	10.854	10.909

**Table 2:** Summary of state–space model and Simple Recurrent Neural Network definition.

Simple Recurrent Neural Network	State-Space Model
$q_{k}=\sigma_{q}\left(Lq_{k-1}+Ep_{k}+z\right)$	$x_{k}=\tilde{A}x_{k-1}+\tilde{B}u_{k-1}$
$v_{k}=\sigma_{u}\left(Pq_{k}+g\right)$	$y_{k}=Cx_{k}$

## Data Availability

Data presented in this study are openly available in FigShare at 10.6084/m9.figshare.20968492, reference number 20968492.

## Supplementary Materials

The following supporting information can be downloaded at: https://www.mdpi.com/article/10.3390/polym14235290/s1, Figure S1: Predicted and real reference outputs of the SRNN, GRU, and LSTM architectures.
