# Supplementary material for: Predictive Modeling of Soft Stretchable Nanocomposites Using Recurrent Neural Networks
Source: Polymers (Basel). 2022 Dec 3;14(23):5290. doi: 10.3390/polym14235290 (PMC9740639; doi:10.3390/polym14235290)
Supplement: Supplementary file 1 [file polymers-14-05290-s001.zip › polymers-1930127-supplementary.pdf]

Supplementary Materials: Predictive Modeling of Soft Stretchable Nanocomposites Using Recurrent Neural Networks

Josué García-Ávila [1,2], Diego de Jesus Torres Serrato [1,3], Ciro. A. Rodriguez [1,4], Adriana Vargas Martínez [1,4], Erick Ramírez Cedillo [1,4,5,*] and J. Israel Martínez-López [1,4,5,6,*]

1 Tecnologico de Monterrey, Escuela de Ingeniería y Ciencias, Monterrey 64849, Mexico; garcia.josue@tec.mx (J.G.-Á.); a00826986@tec.mx (D.d.J.T.S.); ciro.rodriguez@tec.mx (C.A.R.); adriana.vargas.mtz@tec.mx (A.V.M.)
2 Department of Mechanical Engineering, Stanford University, Stanford, CA 94305-2004, USA; jgarciaa@stanford.edu
3 DTU Nanolab, National Centre for Nano Fabrication and Characterization, Technical University of Denmark, 2800 Kgs. Lyngby, Denmark; s222362@dtu.dk
4 Laboratorio Nacional de Manufactura Aditiva y Digital MADiT, Apodaca 66629, NL, Mexico;
5 3D Factory, Ramon Treviño 1109, Monterrey 64580, Mexico
6 Centro de Investigación Numericalc, 5 de mayo 912 Oriente, Monterrey 64000, NL, Mexico
* Correspondence: erickramce@tec.mx (E.R.C.); israel.mtz@tec.mx (J.I.M.-L.)

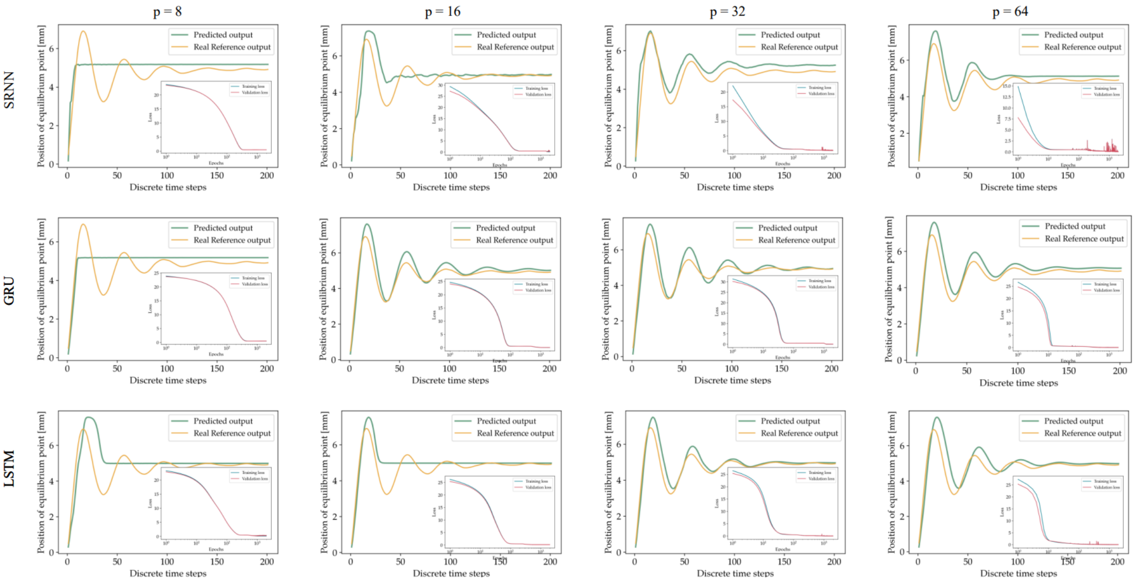

Figure S1. Predicted and real reference outputs of the SRNN, GRU, and LSTM architectures with their training, and validation losses for different number of unitsp.
